# Contrasting genetic metrics and patterns among naturalized rainbow trout (*Oncorhynchus mykiss*) in two Patagonian lakes differentially impacted by trout aquaculture

**DOI:** 10.1002/ece3.3574

**Published:** 2017-11-28

**Authors:** Cristian B. Canales‐Aguirre, Lisa W. Seeb, James E. Seeb, María I. Cádiz, Selim S. Musleh, Ivan Arismendi, Gonzalo Gajardo, Ricardo Galleguillos, Daniel Gomez‐Uchida

**Affiliations:** ^1^ Genomics in Ecology, Evolution and Conservation Lab (GEECLAB) Departamento de Zoología Universidad de Concepción Concepción Chile; ^2^ Laboratorio de Genética y Acuicultura Departamento de Oceanografía Facultad de Ciencias Naturales y Oceanográficas Universidad de Concepción Concepción Chile; ^3^ Nucleo Milenio INVASAL Concepción Chile; ^4^ School of Aquatic and Fishery Sciences University of Washington Seattle WA USA; ^5^ Department of Fisheries and Wildlife Oregon State University Corvallis OR USA; ^6^ Laboratorio de Genética, Acuicultura & Biodiversidad Universidad de Los Lagos Osorno Chile; ^7^ Centro i‐mar Universidad de Los Lagos Camino Chinquihue 6 km Puerto Montt Chile

**Keywords:** aquaculture escapes, Chile, invasion genetics, northern Patagonia, propagule pressure, South America

## Abstract

Different pathways of propagation and dispersal of non‐native species into new environments may have contrasting demographic and genetic impacts on established populations. Repeated introductions of rainbow trout (*Oncorhynchus mykiss*) to Chile in South America, initially through stocking and later through aquaculture escapes, provide a unique setting to contrast these two pathways. Using a panel of single nucleotide polymorphisms, we found contrasting genetic metrics and patterns among naturalized trout in Lake Llanquihue, Chile's largest producer of salmonid smolts for nearly 50 years, and Lake Todos Los Santos (TLS), a reference lake where aquaculture has been prohibited by law. Trout from Lake Llanquihue showed higher genetic diversity, weaker genetic structure, and larger estimates for the effective number of breeders (*N*_b_) than trout from Lake TLS. Trout from Lake TLS were divergent from Lake Llanquihue and showed marked genetic structure and a significant isolation‐by‐distance pattern consistent with secondary contact between documented and undocumented stocking events in opposite shores of the lake. Multiple factors, including differences in propagule pressure, origin of donor populations, lake geomorphology, habitat quality or quantity, and life history, may help explain contrasting genetic metrics and patterns for trout between lakes. We contend that high propagule pressure from aquaculture may not only increase genetic diversity and *N*_b_ via demographic effects and admixture, but also may impact the evolution of genetic structure and increase gene flow, consistent with findings from artificially propagated salmonid populations in their native and naturalized ranges.

## 1 INTRODUCTION

Different pathways of propagation and dispersal of non‐native species into new environments may have contrasting demographic and genetic impacts on established populations (Wilson, Dormontt, Prentis, Lowe, & Richardson, 2009). On the one hand, cultivation of non‐native species may unintentionally release large numbers of individuals in single or multiple events which can be a key factor counteracting detrimental effects of genetic drift among founding populations (Dlugosch & Parker, 2008; Frankham, 2005; Roman & Darling, 2007). These populations will show similar or higher levels of genetic diversity than their native counterparts because propagules may originate from multiple, genetically distinct source populations (Kolbe et al., 2004) or because propagule size is large enough to have high genetic diversity (Simberloff, 2009). On the other hand, human‐driven dispersal (e.g., stocking) involving fewer propagules or propagules of smaller size than in cultivation settings will result in established populations showing lower genetic diversity (Kawamura et al., 2010; Kinziger, Nakamoto, Anderson, & Harvey, 2011; Lindholm et al., 2005). A comparison between these two pathways may reveal contrasting genetic metrics and patterns, including genetic diversity, the evolution of genetic structure and dispersal, and other parameters such as the effective number of breeders (*N*_b_), which likely stem from differences in propagule pressure (Colautti, Grigorovich, & MacIsaac, 2006; Lockwood, Cassey, & Blackburn, 2005; Simberloff, 2009).

Historical introductions of salmonids to South America have been widely documented (Basulto, 2003) and are a useful model to address how invasive populations colonize new environments (Garcia de Leaniz, Gajardo, & Consuegra, 2010; Pascual et al., 2009). Rainbow trout *Oncorhynchus mykiss* Walbaum, 1972 is one of the most broadly and successfully introduced species in freshwater due to its economic value, ease of domestication, and importance in supporting recreational fisheries (Casal, 2006; Crawford & Muir, 2008; Halverson, 2010). Rainbow trout is considered among the world's 100 worst invasive, non‐native species (Lowe, Browne, Boudjelas, & De Poorter, 2000). The species exhibits several life history forms, including fluvial, ad‐fluvial, and anadromous (“steelhead salmon”). These forms may exhibit variable age at maturity, and, unlike many Pacific salmon species, rainbow trout may reproduce multiple times throughout their lifetime (Quinn, 2005). High phenotypic plasticity among rainbow trout has likely resulted in worldwide successful colonization, including most rivers and lakes from the extensive area of Patagonia in South America and particularly Chile (Arismendi et al., 2014; Crawford & Muir, 2008; Soto et al., 2006).

**Figure 1:**
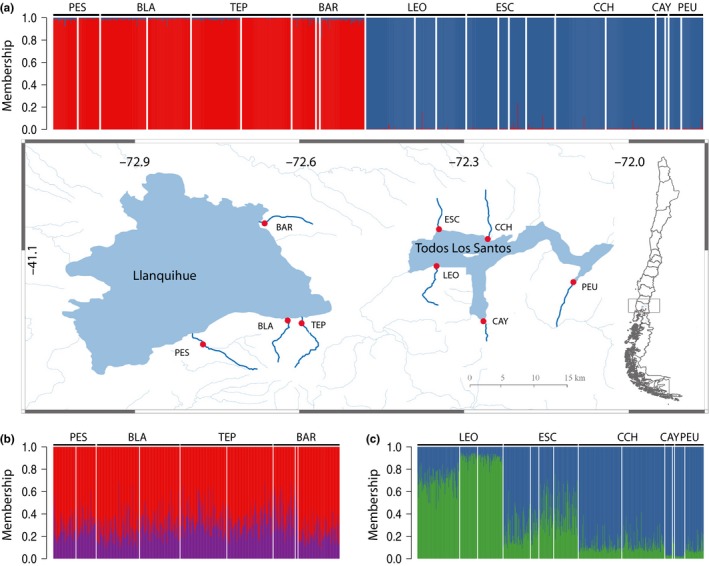
Sampling sites for inlet streams from Lake Llanquihue and Lake Todos Los Santos. Streams sampled in Lake Llanquihue were (from south to north, counterclockwise) Pescado (PES), Blanco (BLA), Tepu (TEP), and Blanco Arenales (BAR). Streams sampled in Lake Todos Los Santos were (from east to west) Leon (LEO), Escape (ESC), Cachimba (CCH), Cayetue (CAY), and Peulla (PEU). (a) Stacked vertical bars represent a vector of membership coefficients (*Q*‐values) of individual genotypes to the most likely number of gene pools (*K* = 2) using data from 86 SNPs, (b) within Lake Llanquihue only (*K* = 2), (c) within Lake Todos Los Santos only (*K* = 2). White vertical lines separate year or season collections within inlet streams. Name of each inlet stream is located above the bar graph

The first introductions of rainbow trout to the Lake District in Chile's northern Patagonia supported recreational fisheries in the late 1890s and early 1900s (Basulto, 2003). Further introductions occurred at the onset of the trout aquaculture industry during the late 1970s and development throughout the 1980s following unintentional escapes (Basulto, 2003; Garcia de Leaniz et al., 2010). Lake Llanquihue and Lake Todos Los Santos (TLS) are two Araucanian lakes situated in the northern Patagonia (Figure 1) where these invasion pathways can be studied; each lake provides contrasting histories of trout introductions, especially with respect to the influence of trout aquaculture. The lakes further differ in origin, size, and geomorphology (Table 1). On the one hand, Basulto (2003) reported that Lake Llanquihue was stocked early during the twentieth century and later influenced by aquaculture escapes (Arismendi et al., 2009). Lake Llanquihue has been Chile's largest producer of salmonid smolts for the aquaculture industry since 1975; between 1998 and 2005 Lake Llanquihue produced 5.1 million rainbow trout smolts (León‐Muñoz, Tecklin, Farias, & Diaz, 2007). Estimates suggest that 3–5% of fish production from net pens are accidentally released into the environment every year through an undocumented number of escapes (Sepúlveda, Arismendi, Soto, Jara, & Farias, 2013). Rainbow trout escapes are likely to become rapidly established in the wild (Arismendi et al., 2014; Benavente et al., 2015). On the other hand, Lake TLS is located within “Vicente Perez Rosales” National Park and Reserve, wherein aquaculture has been prohibited by law. However, Basulto (2003) reported one trout stocking event in the eastern shore of Lake TLS during the early 1900s. A second introduction of unknown location occurred around 1980 (Arismendi et al., 2009), suggesting Lake TLS has received fewer propagules than Lake Llanquihue. The latter introduction appears to be the source of currently abundant naturalized, self‐sustaining trout populations (Arismendi et al., 2009).

**Table 1:** Lake Llanquihue and Lake Todos Los Santos physical attributes and summary of invasion pathways for rainbow trout

	Lake Llanquihue	Lake Todos Los Santos	References
Physical attributes			
Water surface area (km^2^)	870.5	178.5	Campos et al. (1988, 1990)
Elevation (m)	51	189	Campos et al. (1988, 1990)
Origin	Glacial	Volcanic	Campos et al. (1988, 1990)
Water volume (km^3^)	158.6	34.4	Campos et al. (1988, 1990)
Catchment area (km^2^)	1605	3036	Campos et al. (1988, 1990)
Invasion pathways			
Stocking versus aquaculture	Stocking: 1910–1916, temporary hatchery placed at the outlet (River Maullín) that maintained 50,000–100,000 eggs imported from Germany	Stocking: 1932, anglers transplanted an unknown number of individuals at Negro River near Peulla, the easternmost location of the lake	Arismendi et al. (2009); Basulto (2003); Benavente et al. (2015); León‐Muñoz et al. (2007); Sepúlveda et al. (2013)
	Aquaculture: 1969–1972, first aquaculture farm that bred 37,500 adults and reported “massive” escapes	Stocking: ~1980, anglers transplanted an unknown number of individuals at western locations of the lake	
	Aquaculture: 1975–1979, second aquaculture farm near River Pescado yielding 40,000 kg annually for exportation to France		
	Aquaculture: 1980–2017, consolidated industry with 15 companies yielding 5.1 million smolts annually (1998–2005). Escapes of nearly 500,000 trout in lakes, including Lake Llanquihue (2004–2012)		

Genetic assessments have been instrumental in evaluating the geographic origin as well as drivers that explain the establishment and spread of naturalized rainbow trout in Patagonia (Gajardo, Díaz, & Crespo, 1998; Monzón‐Argüello, Garcia de Leaniz, Gajardo, & Consuegra, 2013, 2014; Monzón‐Argüello, Consuegra, et al., 2014; Riva Rossi, Lessa, & Pascual, 2004). Recently, Benavente et al. (2015) successfully used single nucleotide polymorphisms (SNPs) developed from a panel of North American populations to estimate intra‐ and interpopulation divergence among rainbow trout in Lake Llanquihue. Naturalized rainbow trout populations harbored high genetic diversity, providing an important molecular resource for invasion genetics studies; putative escaped trout were also identified (Benavente et al., 2015). Highly variable SNPs may allow fine‐scale resolution of population differences where other markers have failed to resolve them (Ackerman, Habicht, & Seeb, 2011; Aykanat et al., 2015; Hecht, Campbell, Holecek, & Narum, 2013; Zarraonaindia et al., 2012).

A comparison among rainbow trout populating Lake Llanquihue and Lake TLS provides a unique opportunity to study the genetic underpinnings of two different invasion pathways. Adult rainbow trout of ad‐fluvial life history breed at inlet streams of Araucanian lakes; hatched juveniles may then spend 1–2 years before migrating to feed in the lake (Arismendi, Sanzana, & Soto, 2011). Here, we tested several expectations about trout genetic metrics and patterns in both lakes (Table 2). We hypothesized that trout from inlet streams in Lake Llanquihue will show higher genetic diversity, weaker genetic structure, and larger estimates of *N*_b_ compared to rainbow trout collected from inlet streams in Lake TLS. Increased gene flow resulting from admixture has been observed among experimental colonizing (Szűcs, Melbourne, Tuff, & Hufbauer, 2014) and salmonid populations in their native (Boyer, Muhlfeld, & Allendorf, 2008; Candy & Beacham, 2000) and naturalized ranges (Bartron & Scribner, 2004). In addition, multiple introductions via artificial propagation may positively impact effective population size and related measures such as *N*_b_ (Præbel, Gjelland, Salonen, & Amundsen, 2013).

**Table 2:** Expected relative values of genetic metrics and patterns among naturalized rainbow trout

Population genetics metrics	Relative expected value	
	Lake Llanquihue	Lake Todos Los Santos
Genetic diversity	High	Low
Genetic structure	Weak	Moderate
Effective number of breeders	Large	Small

Values were based on differences on invasion pathways and propagule pressure between Lake Llanquihue and Lake Todos Los Santos.

## 2 MATERIAL AND METHODS

### 2.1 Sampling design

A total of 1,193 naturalized rainbow trout were sampled between 2013 and 2014 from inlet streams of Lake Llanquihue and Lake TLS (Figure 1, Table 3). Inlet streams of Lake Llanquihue are found only on the west and south shores. Samples taken during 2013 at Lake Llanquihue were from the Benavente et al. (2015) study after excluding one inlet stream likely populated by escaped trout (i.e. Pescado (PES), Blanco (BLA), Tepu (TEP), and Blanco Arenales (BAR)). Additionally, we sampled five inlet streams from Lake TLS: León (LEO), Escape (ESC), Cachimba (CCH), Cayetue (CAY), and Peulla (PEU) (Figure 1). All individuals were collected during two austral seasons: winter, spring, or both, corresponding to the spawning period of rainbow trout in this region (Arismendi et al., 2011). Collections were made with a two‐pass backpack electrofishing using various settings depending on water conductivity (400–700 V; 40–80 Hz). Within each inlet stream, we sampled a 400 m reach with a uniform time effort of 1 h/stream. To account for fish of different sizes, we sampled in all available habitat units (pool‐run‐riffle). Nonlethal sampling of fin clips of each individual was taken and preserved in ethanol 95% for further genetic analyses.

**Table 3:** Parameters of genetic diversity among naturalized rainbow trout collected at inlet streams from Lake Llanquihue and Lake Todos Los Santos

Lake	Stream	Sample date	Code	*N*	*N*_A_	*H*_O_	*H*_E_	*f*	*A*_R_
Llanquihue	PES	July 2013	PES13W	46	1.965	0.320	0.317	−0.014	1.690
		October 2014	PES14S	40	1.988	0.325	0.33	0.016	1.717
	BLA	July 2013	BLA13W	88	1.988	0.320	0.324	0.007	1.700
		October 2014	BLA14S	82	2.000	0.310	0.329	0.058	1.708
	TEP	July 2013	TEP13W	95	1.988	0.344	0.33	−0.037	1.71
		October 2014	TEP14S	94	1.977	0.351	0.329	−0.052	1.707
	BAR	July 2013	BAR13W	44	2.000	0.340	0.341	−0.003	1.733
		October 2014	BAR14S	85	1.988	0.299	0.319	0.05	1.688
Todos Los Santos	LEO	July 2013	LEO13W	93	1.791	0.260	0.252	−0.027	1.542
		July 2014	LEO14W	38	1.767	0.259	0.253	−0.027	1.551
		October 2014	LEO14S	55	1.779	0.275	0.26	−0.043	1.560
	ESC	July 2013	ESC13W	59	1.791	0.242	0.248	0.020	1.542
		Oct 2013	ESC13S	17	1.709	0.247	0.241	−0.029	1.538
		July 2014	ESC14W	31	1.849	0.277	0.268	−0.008	1.591
		October 2014	ESC14S	53	1.826	0.262	0.258	−0.017	1.564
	CCH	July 2013	CCH13W	95	1.802	0.244	0.247	0.009	1.534
		October 2014	CCH14S	94	1.814	0.252	0.248	−0.011	1.541
	CAY	July 2013	CAY13W	15	1.756	0.253	0.244	−0.031	1.548
	PEU	July 2013	PEU13W	21	1.756	0.255	0.246	−0.034	1.549
		October 2014	PEU14S	41	1.791	0.246	0.243	0.000	1.534

*N = *sample size*, N*_A_ = number of alleles*, H*_O_ = observed heterozygosity*, H*_E_ = expected heterozygosity*, f = *inbreeding coefficient*, A*_R_ = allelic richness. Location name (three capital letters) is followed by year (i.e., 2013 = 13 or 2014 = 14) and season (i.e., winter = W or spring = S).

### 2.2 Molecular procedures, exploratory analyses, and SNP selection

Genomic DNA was isolated using a NucleoSpin^®^ (Macherey‐Nagel) kit following the manufacturer's instructions. We amplified 96 SNPs (Table S1) that yielded polymorphic multilocus genotypes in both native (Jones et al., 2015) and naturalized rainbow trout populations (Benavente et al., 2015) (Table S2). Multiplex PCR was carried out using Fluidigm^®^ 96.96 dynamic array chips following Seeb, Pascal, Ramakrishnan, and Seeb (2009) and procedures of Smith et al. (2011) for a pre‐amplification step to increase PCR copies under low template DNA concentration.

We conducted exact tests using default Markov chain parameters in GENEPOP 3.1 (Raymond & Rousset, 1995; Rousset, 2008) for testing deviations from Hardy–Weinberg equilibrium (HWE) proportions and linkage disequilibrium (LD) among loci. We filtered out all loci that showed significant deviations from HWE (*p* < .05). SNPs with the lowest information content within each group of loci in LD were also excluded. Information content was gauged by Shannon–Weaver index calculated in GENALEX 6.5 software (Peakall & Smouse, 2012).

We additionally explored the presence of siblings that may have occurred within our collections and indicative of family‐biased sampling, a concern that has motivated scientists to purge related individuals (Waples & Anderson, 2017). Maximum likelihood estimates of relatedness among individuals were obtained using ML‐Relate (Kalinowski, Wagner, & Taper, 2006) in order to classify individuals as unrelated, half‐sibs or full‐sibs within inlet stream collections for each lake.

### 2.3 Hierarchical analysis of molecular variance (AMOVA)

We conducted a hierarchical analysis of molecular variance (AMOVA; Excoffier, Smouse, & Quattro, 1992) in ARLEQUIN v3.5 (Excoffier & Lischer, 2010) to evaluate the magnitude of spatial versus temporal variation and whether year collections from the same stream (i.e., year or season) should be pooled or kept separate. Hierarchical groups were variance components between year or season collections within streams (intrapopulation; *F*_SC_) and between streams (interpopulation; *F*_CT_) following Benavente et al. (2015).

### 2.4 Genetic diversity

We tested for significant differences between lakes on observed (*H*_O_) and expected heterozygosities (*H*_E_) and allelic richness (*A*_R_). Calculation of *H*_O_ and *H*_E_ was conducted in GENALEX; *A*_R_ was estimated in HierFstat v0.04‐10 package (de Meeûs & Goudet, 2007) implemented in R (R Core Team, 2015). Inbreeding coefficients (*f*) to evaluate deficit or excess of heterozygotes on deviations from HWE proportions were calculated in GENALEX. We built boxplots for each genetic diversity index and inlet stream within lakes and tested for statistical differences (*p* < .05) between lakes using the nonparametric Mann–Whitney *U* test in R.

### 2.5 Genetic population structure and divergence within and between lakes

We employed three approaches to infer genetic divergence: (i) theta (*θ*_ST_, Weir & Cockerham, 1984) using GENEPOP, (ii) a Bayesian approach based on genetic clustering methods using STRUCTURE v.2.3.3 software (Falush, Stephens, & Pritchard, 2003; Pritchard, Stephens, & Donnelly, 2000), and (iii) discriminant analysis of principal components (DAPC) as part of ADEGENET v1.3‐1 for R (Jombart, 2008; Jombart & Ahmed, 2011; Jombart, Devillard, & Balloux, 2010). To estimate pairwise *θ*_ST_ values, we compared inlet streams between the two lakes and within each lake separately. To estimate significant differentiation between samples, we conducted Fisher's exact probability test (Markov chain parameters: dememorization number = 1,000, number of batches = 100 and number of iterations per batch = 10,000) in GENEPOP. Using STRUCTURE, we tested *K* values ranging from one to five, with 50,000 iterations of burn‐in and a run length MCMC of 250,000; all these runs were replicated ten times. These runs were conducted using an ancestry mixture model and locality information priors to improve the detection of structure when genetic structure is weak (Hubisz, Falush, Stephens, & Pritchard, 2009). We conducted this analysis for the entire dataset (both lakes) and by lake. We plotted “consensus” coefficients of individual membership using an R code following cluster matching and permutation in CLUMPP software (Jakobsson & Rosenberg, 2007) to account for label switching artifacts and multimodality in each *K* tested. To choose the most likely *K* value from these analyses, we conducted the Δ*K* Evanno's index (Evanno, Regnaut, & Goudet, 2005) implemented in STRUCTURE HARVESTER website (Earl & vonHoldt, 2012). Finally, we used DAPC to estimating and plotting individual pairwise genetic distances. DAPC reduces multivariate SNP multilocus data into two orthogonal axes and ignores assumptions (e.g., HWE, LD) often required in other individual‐based models.

We used a Mantel test to correlate standardized genetic (*θ*_ST_/[1 − *θ*_ST_]) and geographic distances to test for isolation by distance between inlet streams within each lake. The Mantel test was conducted in *ade4* software package based on 100,000 permutations (Chessel, Dufour, & Thioulouse, 2004; Dray & Dufour, 2007; Dray, Dufour, & Chessel, 2007; Thioulouse, Chessel, Dolédec, & Olivier, 1997). Pairwise genetic distances were plotted against coastwise distances along lake shores (in km) that separated inlet streams estimated in QGIS (QGIS Development Team 2016), an open‐access geographic information system.

### 2.6 Linkage disequilibrium estimates for the effective number of breeders (*Nb*)

We used Waples (2006) method to estimate effective population size, which measures LD between unlinked loci to approximate genetic drift under the following assumptions: selective neutrality, discrete generations, and closed populations. Because rainbow trout have overlapping generations, LD reflects a quantity closer to *N*_b_ per brood year rather than the effective population size (Waples & Do, 2010). LD method is also robust to violations of the assumption of closed populations, granted migration rates are lower than 5% (Waples & England, 2011). We estimated LD *N*_b_ using a threshold frequency of 0.01 for screening out rare alleles in NeEstimator v2.0 software (Do et al., 2014); we also assumed random mating, and 95% CIs for *N*_b_ were calculated using jackknifing among pairs of loci. A Mann–Whitney *U* test in R was implemented to test for significant differences in LD *N*_b_ between lakes.

## 3 RESULTS

### 3.1 Exploratory analyses and SNP selection

We excluded one locus that deviated from HWE (*Omy_mcsf_268‐A1*), six loci that were found in LD with more informative markers (*OMS00012, Omy_arp‐630, Omy_nkef‐308, Omy_dacd1‐131, Omy_U11_2a‐114, OMS00177*), and three monomorphic markers (*Ocl_Okerca, Ocl_oku202, Ocl_Oku216*). This filtering procedure yielded a set of 86 reliable SNPs for subsequent analyses (Table S1).

We found no evidence for large numbers of siblings that may bias estimates of population differentiation or inference of genetic structure as all collections contained >85% of unrelated individuals. Also, the proportion of related individuals (half‐ and full‐sibs) varied little across collections (10–15%). We thus opted for retaining related individuals as the incidence of family structure was low (Waples & Anderson, 2017).

### 3.2 Hierarchical AMOVA

We found small but significant temporal variance among collections within streams for both lakes (Table 4): Lake Llanquihue (*F*_SC_ = 0.004, *p* < .001) and Lake TLS (*F*_SC_ = 0.006, *p* < .001). Spatial (interpopulation) variance was greater for rainbow trout from Lake TLS (*F*_CT_ = 0.012, *p* = .0009) than from Lake Llanquihue (*F*_CT_ = 0.001, *p* = .075). Based on these results, we followed Benavente et al. (2015) approach and kept all collections separate and considered them independent for subsequent tests involving contrasts between lakes.

**Table 4:** Hierarchical analysis of molecular variance (AMOVA) to test for spatial versus temporal variation among naturalized rainbow trout in two Patagonian lakes

Hierarchical groups	% of variation	*F*‐statistics	*p*‐value
Lake Llanquihue			
Between streams	0.13	*F*_CT_ = 0.001	.075
Between collections within streams	0.41	*F*_SC_ = 0.004	<.001
Lake Todos Los Santos			
Between streams	1.23	*F*_CT_ = 0.012	.0009
Between collections within streams	0.62	*F*_SC_ = 0.006	<.001

### 3.3 Genetic diversity

**Figure 2:**
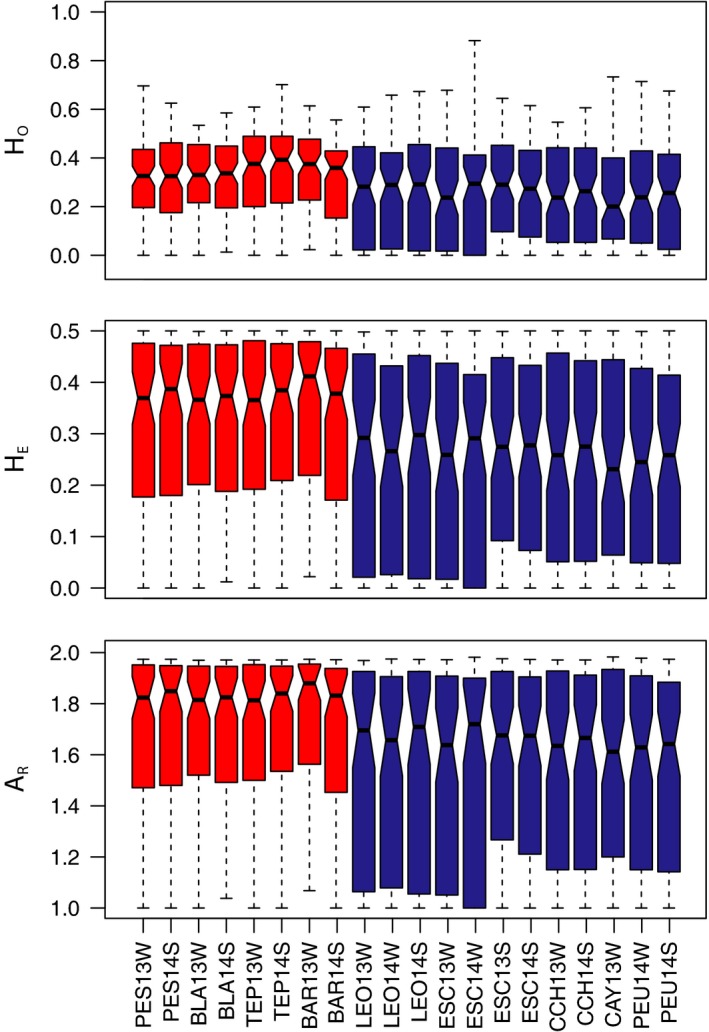
Boxplots of genetic diversity for rainbow trout captured at inlet streams in two northern Patagonian lakes (Lake Llanquihue = red boxes; Lake Todos Los Santos = blue boxes). The upper panel shows observed heterozygosities (*H*_O_), the middle panel shows expected heterozygosities (*H*_E_), and the lower panel shows allelic richness (*A*_R_)

Range of values for all three parameters—*H*_O_, *H*_E_ and *A*_R_—differed between Lake Llanquihue and Lake TLS (Figure 2). *H*_O_ ranged between 0.299 and 0.351 for Lake Llanquihue and between 0.242 and 0.277 for Lake TLS; *H*_E_ between 0.317 and 0.341 for Lake Llanquihue and between 0.241 and 0.268 for Lake TLS; and *A*_R_ ranged between 1.688 and 1.717 for Lake Llanquihue and between 1.534 and 1.591 for Lake TLS (Table 3). Mean values for all parameters were greater for rainbow trout from Lake Llanquihue than from Lake TLS (all Mann–Whitney *U* tests, *p* < .001). Inbreeding coefficients *f* showed no evidence for heterozygote deficit or excess and were consistent with fit to HWE proportions within collections.

### 3.4 Genetic population structure and divergence between and within lakes

**Figure 3:**
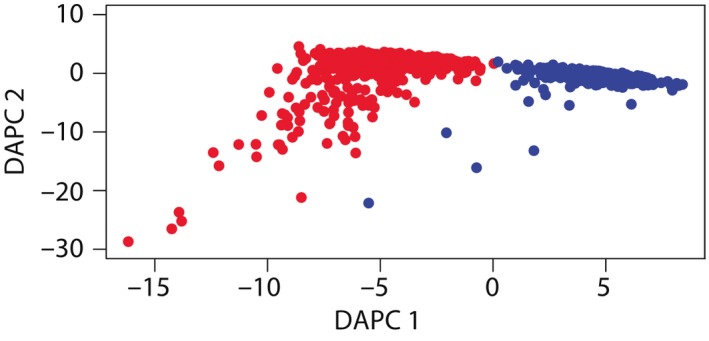
Scatterplot of individual rainbow trout genotypes from discriminant analyses of principal components sampled from Lake Llanquihue (red circles) and Lake Todos Los Santos (blue circles). Genotypes were probabilistically assigned to two clusters

Pairwise *θ*_ST_ ranged between −0.022 and 0.156; the strongest divergence was found among rainbow trout from inlets located in different lakes (*θ*_ST_ > 0.1: Table S3). Results from STRUCTURE assignment, based on Δ*K* Evanno's index, strongly supported *K* = 2 for the entire dataset (Figure 1a). Separate analyses for each lake indicated that rainbow trout genotypes from Lake Llanquihue comprise one cluster (Figure 1b); conversely, we found evidence for two genetic clusters within Lake TLS (Figure 1c). Genetic clusters were consistent with an eastern–western dichotomy: inlet streams CCH‐CAY‐PEU versus LEO, with ESC showing intermediate *Q*‐values to each cluster. DAPC confirmed strong differentiation by lake (Figure 3).

**Figure 4:**
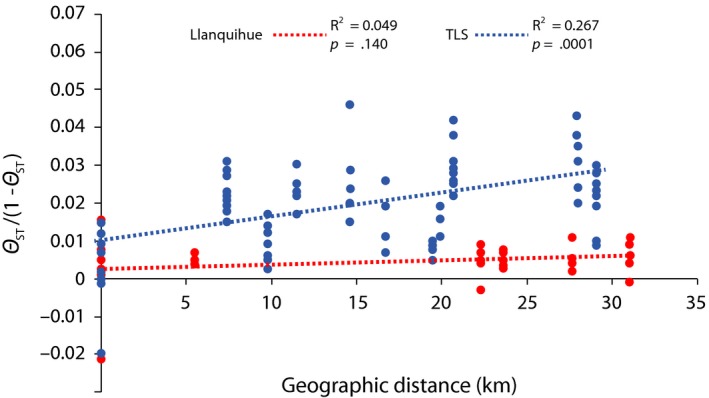
Relationship between linearized genetic (*ϴ*_ST_/[1 − *ϴ*_ST_]) and geographic distances among rainbow trout collections from Lake Llanquihue (red circles) and Lake Todos Los Santos (blue circles). Determination coefficients (*R* ^2^) and *p*‐values for regression models are shown above the graph

Patterns of isolation by distance differed significantly between lakes. Rainbow trout from Lake Llanquihue showed no significant relationship between genetic and geographic distances, whereas rainbow trout from Lake TLS exhibited a significant relationship (Figure 4; *R* ^2^ = 0.267; *p* = .001).

### 3.5 Effective number of breeders from linkage disequilibrium (*N*_b_)

Estimates of LD *N*_b_ were larger for rainbow trout from Lake Llanquihue than from Lake TLS (Table 5; Mann–Whitney *U* test = 85; *p*‐value <.01). All point estimates were finite and bound by finite 95% CIs. Values for LD *N*_b_ ranged between 86 and 223 individuals (average = 139; median = 126) for Lake Llanquihue and between 13 and 164 individuals (average = 55; median = 40) for Lake TLS.

**Table 5:** Linkage disequilibrium (LD) estimates (plus 95% CI) for the effective number of breeders for rainbow trout populating inlet streams of Lake Llanquihue and Lake Todos Los Santos

Lake	Inlet stream	LD *N*_b_	95% CI
Llanquihue	PES13W	121	79.6–228.2
	PES14S	187	100.7–864.5
	BLA13W	98	78.9–126.8
	BLA14S	147	107–222.5
	TEP13W	119	94.4–157.7
	TEP14S	132	100.6–183.1
	BAR13W	86	61.6–132.9
	BAR14S	223	146.9–423.9
Todos Los Santos	LEO13W	48	40.3–57.3
	LEO14W	47	34.6–69.1
	LEO14S	34	27.7–42.7
	ESC13W	67	50.5–93.7
	ESC14W	19	15.5–24.1
	ESC13S	30	18.2–63.2
	ESC14S	29	24.2–35.6
	CCH13W	139	101.3–208.2
	CCH14S	164	115.7–261.2
	CAY13W	13	9.2–18.8
	PEU14W	23	16.4–34.8
	PEU14S	46	34.6–65.8

Location names (three capital letters) correspond to inlet stream abbreviations followed by two‐digit years (2013, 2014) and season (winter = W, spring = S).

## 4 DISCUSSION

We evaluated how different pathways of rainbow trout invasion, exemplified by two Patagonian lakes with and without the influence of trout aquaculture, have resulted in differences in population genetics metrics and patterns such as diversity, structure, divergence, and effective number of breeders (LD *N*_b_). Using a suite of 86 SNPs, we found higher genetic diversity and larger estimates of number of breeders in Lake Llanquihue, a lake heavily influenced by aquaculture escapes and subject therefore to higher propagule pressure than Lake TLS. Genetic structure and a significant isolation‐by‐distance pattern were evident in Lake TLS, but not in Lake Llanquihue, in line with secondary contact between two introductions in opposite shores of the lake. Divergence between lakes was also large, suggesting trout inhabiting these two lakes originated from different donor populations.

### 4.1 Genetic diversity

Genetic diversity parameters (i.e., *H*_O_, *H*_E_, *A*_R_) were higher for trout from Lake Llanquihue than from Lake TLS. We hypothesize that high propagule pressure from late aquaculture escapes, which followed an early and brief stocking phase, explain these differences. From 1910 to 1916, between 50,000 and 100,000 rainbow trout eggs were imported from Germany and maintained in a temporary hatchery located at the outlet of Lake Llanquihue, Maullin River, for stocking purposes (Basulto, 2003). The German stocks originated from hatcheries from Northville, Michigan, and Wytheville, Virginia; the Michigan and Virginia stocks were derived from Baird Station at McCloud River, California (Stanković, Crivelli, & Snoj, 2015), suggesting rainbow trout from Lake Llanquihue can also be traced back to their native origin (MacCrimmon, 1971). Pilot aquaculture projects (1969–1979) followed, which reported “massive” escapes (Basulto, 2003), and subsequent rapid growth of the aquaculture industry in freshwater lakes after 1980 (Arismendi et al., 2014). Currently, Lake Llanquihue concentrates the highest number of aquaculture facilities among lakes in the northern Patagonia region (León‐Muñoz et al., 2007). Escapes of salmonids could be in the order of million individuals annually, with rainbow trout possibly reaching hundreds of thousands of escaped individuals, especially in freshwater lakes, though disaggregated data by location are lacking (Sepúlveda et al., 2013).

High genetic diversity linked to propagule pressure may be a consequence of demographic effects such as high abundance, genetic effects such as admixture between divergent sources (Roman & Darling, 2007; Simberloff, 2009), or both. First, Arismendi et al. (2009) estimated one the highest abundances of rainbow trout in Lake Llanquihue among several other lakes impacted by trout aquaculture. Second, Consuegra, Phillips, Gajardo, and Garcia de Leaniz (2011) reported that admixture between naturalized and aquaculture‐escaped rainbow trout increased genetic diversity, and Benavente et al. (2015) demonstrated that aquaculture‐escaped rainbow trout often have higher genetic diversity than naturalized, and even native, rainbow trout populations. These studies support the notion that an invasion pathway originating from unintentional escapes from aquaculture, and similar practices among cultivable species, could enhance genetic diversity and reduce genetic drift via increased propagule pressure (Simberloff, 2009; Wilson et al., 2009).

The invasion pathway for rainbow trout within Lake TLS likely resulted from two discrete stocking events, one documented and one undocumented, unlike continuous propagation as hypothesize for Lake Llanquihue. In 1932, anglers stocked Negro River near PEU (Figure 1), the easternmost location of the lake (Basulto, 2003). Additional stocking in western inlet rivers (e.g., LEO) may have occurred mid‐century (R. Yefi, pers. comm. 2016) or around 1980s (Arismendi et al., 2009), though no official records exist. Nonetheless, our results make plausible the hypothesis of more than one introduction in opposite shores of the lake (see next section, Genetic population structure and divergence). Establishment of trout was likely mediated by lower propagule pressure than in the case of Lake Llanquihue, suggesting populations at Lake TLS (i) were founded by a small number of individuals (i.e., founder effect), (ii) underwent genetic bottlenecks postestablishment, or both. Even established non‐native populations of low genetic diversity may drive successful invasions (Chapple, Miller, Kraus, & Thompson, 2013; Dlugosch & Parker, 2008; Kawamura et al., 2010). Rainbow trout that colonized Lake TLS is no exception as they encompass abundant populations (Arismendi et al., 2009; Soto et al., 2006), implying there are yet unanswered questions on the relationship between genetic diversity and invasion success (Barrett, 2015; Bock et al., 2015).

### 4.2 Genetic population structure and divergence

Both population‐ and individual‐based inference from various approaches showed consistent results: genetic divergence was large between rainbow trout populating Lake Llanquihue and Lake TLS. Donor trout populations were likely different between lakes, though this hypothesis is difficult to test given no background information on trout origin for Lake TLS is available. Many genetically distinct broodstocks of rainbow trout were imported to Chile for aquaculture (Cárcamo, Díaz, & Winkler, 2015), and hybridization between varieties and lineages was a common hatchery practice. Additionally, hybridization between aquaculture and naturalized trout may have occurred in the wild as explored by previous studies (Benavente et al., 2015; Consuegra et al., 2011). These factors may explain the observed genetic differences between lakes, which were possibly further enhanced by a strong founder effect and genetic drift among rainbow trout from Lake TLS.

Within lakes, we found contrasting patterns of genetic structure; for instance, we observed weak genetic structure for rainbow trout within Lake Llanquihue. Native ad‐fluvial trout have evolved significant genetic structure and divergence (Knudsen, Muhlfeld, Sage, & Leary, 2002; Leitwein, Garza, & Pearse, 2017), and other naturalized ad‐fluvial trout have also shown significant genetic differentiation (Krueger & May, 1987). Weak genetic structure in Lake Llanquihue rainbow trout may result from increased genetic diversity via admixture, which may in turn increase dispersal rates (and thus expansion) among salmonid populations. Bartron and Scribner (2004) reported weak genetic structure as well as temporal changes in genetic diversity and spatial genetic structure as a result of hatchery supplementation on Lake Michigan naturalized steelhead salmon populations. Candy and Beacham (2000) used coded‐wire tagged Chinook salmon from British Columbia populations to conclude that a stock of hybrid origin was three times more likely to disperse than a natal stock released at the same location. Boyer et al. (2008) demonstrated that hybrids between invasive rainbow and native cutthroat show increased straying rates, which may facilitate rainbow trout invasion and introgression between these two taxa. Both phenotypic and behavioral changes induced by admixture and introgression resulting from aquaculture practices may influence gene flow and thus alter genetic structure (Bolstad et al., 2017; Perrier, Guyomard, Bagliniere, Nikolic, & Evanno, 2013; Waples, 1991).

In contrast, rainbow trout in Lake TLS showed larger genetic divergence than in Lake Llanquihue, evidence for two genetic clusters, and a pattern of isolation by distance. Increased genetic drift from propagules of small size may provide more opportunity for divergence to occur following successful establishment (Kawamura et al., 2010; Roman & Darling, 2007). The presence of two clusters suggests that more than one introduction from at least two different lineages occurred in Lake TLS, though the actual numbers of stocking events and distinct donor populations are uncertain. Our finding is consistent with two stocking events (i.e., documented and undocumented, Table 1) in the eastern and western shores of Lake TLS and the most divergent inlet streams: LEO and PEU (Figure 1). Secondary contact between feral and farmed populations was behind an isolation‐by‐distance pattern found in non‐native American mink invading Europe (Bifolchi, Picard, Lemaire, Cormier, & Pagano, 2010). We hypothesize that eastern (LEO) and western (PEU) rainbow trout, which were propagated from different introduction events, came into contact and resulted in admixed individuals at some streams located in the middle of Lake TLS, namely ESC.

Lake Llanquihue and Lake TLS differ significantly in geomorphology: the former has convex coast lines that may promote gene flow, while the latter has concave coast lines that may restrict gene flow. Evidence from native salmonids demonstrates that drainage geomorphology may promote or restrict gene flow among populations (Carlsson & Nilsson, 2001; Whiteley, Spruell, & Allendorf, 2004), although much less is known in relation to naturalized salmonids. Love, Maggs, Murray, and Provan (2013) report that gene flow among invasive riparian plants is dependent on river flow and geomorphology, suggesting that habitat physical attributes may rapidly influence genetic structure in colonizing species.

Overall, different invasion pathways of rainbow trout that varied in propagule pressure for Lake Llanquihue and Lake TLS may explain differences in genetic structure and gene flow between these two Patagonian lakes. However, additional sources of variation between lakes are worth discussing, namely origin of donor populations and effects of lake geomorphology on dispersal among ad‐fluvial trout populations. It is also important to notice that we still have a limited understanding of how adaptive divergence unfolds among naturalized rainbow trout populations in different environments, for example with and without influence of aquaculture. Monzón‐Argüello, Consuegra, et al. (2014) found that “secondary releases” (when referring to aquaculture escapes) have driven phenotypic divergence among naturalized rainbow trout from the Lake District in Chile. Recently, Bolstad et al. (2017) evidenced that gene flow from domesticated to wild Atlantic salmon influences sea age at maturity. We therefore encourage further studies relying on size‐related phenotypic and quantitative traits to test whether adaptive divergence among rainbow trout varies between environments with and without the influence of aquaculture.

### 4.3 Effective number of breeders (LD *N*_b_)

Estimates for the effective number of breeders using LD (*N*_b_) were larger for rainbow trout from Lake Llanquihue than from Lake TLS. Estimates of LD *N*_b_ from this study were consistent with Benavente et al. (2015) for individuals sampled during 2012 and 2013 at Lake Llanquihue, although with some nuances based on the fact that Benavente et al. (2015) used 81 SNPs. Results from both studies are consistent with increased genetic drift and significant temporal genetic variance estimated over three years of sampling for trout populations from Lake Llanquihue. Significant temporal variation was also found for trout from Lake TLS.

Our LD *N*_b_ results are in line with findings showing higher genetic diversity for Lake Llanquihue and lower genetic diversity for Lake TLS. Artificially propagated populations resulting from multiple introduction events may show effective population sizes greater than naturally propagated ones as seen in introduced vendace *Coregonus albula* in Europe (Præbel et al., 2013) and brook trout in North America (Neville & Bernatchez, 2013). However, Narum et al. (2017) recently found that estimates of LD *N*_b_ among Chinook salmon *Oncorhynchus tshawytscha* populations introduced in Patagonia were smaller than among native populations, suggesting that there may be exceptions to these findings.

Other factors may also interact with the magnitude of propagule pressure to explaining differences in LD *N*_b_ between rainbow trout inhabiting Lake Llanquihue and Lake TLS. First, differences in habitat quality and quantity between lakes may influence population abundance, and indirectly, LD *N*_b_, as reported in other salmonids (Fraser, Lippé, & Bernatchez, 2004; Gomez‐Uchida, Dunphy, O'Connell, & Ruzzante, 2008). Rainbow trout is the dominant species within Lake Llanquihue, whereas rainbow trout have to coexist with abundant populations of brown trout and native fishes within Lake TLS (Arismendi et al., 2009). Second, life history parameters for rainbow trout may vary between lakes, namely sex ratio, variation in offspring number and family size, and generation length. Third, violation of the assumption of closed populations may also explain differences in trout LD *N*_b_ between lakes. Waples and England (2011) concluded that when migration rates are higher than 5–10%, local estimates of effective population size via LD will converge toward the effective metapopulation size. This could potentially be the case of Lake Llanquihue rainbow trout as we observed weak genetic structure using clustering methods and genetic differentiation was small (*θ*_ST_ < 0.01), suggesting estimates of LD *N*_b_ are potentially converging toward the effective number of breeders in the entire metapopulation, meta‐*N*_b_. Yet, differences in meta‐*N*_b_ for rainbow trout between lakes could persist if meta‐*N*_b_ is much smaller than the sum of local LD *N*_b_, which may occur under gene flow asymmetry between populations (e.g., Gomez‐Uchida, Palstra, Knight, & Ruzzante, 2013).

Small effective population size (and *N*_b_) among native populations has been related to a high risk of extinction from genetic stochasticity (Palstra & Ruzzante, 2008). Although many introduced species undertake identical processes during the establishment phase, they have shown to increase their geographical ranges and successfully invade (Kolbe et al., 2004). An extreme example has been recorded in lake trout *Salvelinus namaycush* with a founding size of only two individuals that established a self‐sustaining population (Kalinowski, Muhlfeld, Guy, & Cox, 2010). Recent findings reinforce the notion that even populations of small effective population size may be influenced by selective regimes in their native (Fraser, Debes, Bernatchez, & Hutchings, 2014) and introduced environments (Kawamura et al., 2010), and that deterministic processes and adaptive divergence may mediate successful invasions even when LD *N*_b_ is small.

## CONFLICT OF INTEREST

None declared.

## DATA ARCHIVING STATEMENT

Data available from the Dryad Digital Repository at https://doi.org/10.5061/dryad.gd844.

## AUTHORS' CONTRIBUTIONS

Conception or design of the work: DG‐U IA LWS JES GG. Acquisition, analysis, or interpretation of data for the work: CBC‐A MIA SSM RG DG‐U. Drafting the work or revising it critically for important intellectual content: CBC‐A LWS JES IA GG SSM DG‐U. Final approval of the version to be published: all authors. Agreement to be accountable for all aspects of the work in ensuring that questions related to the accuracy or integrity of any part of the work are appropriately investigated and resolved: all authors.
