# Supplementary material for: Contrasting genetic metrics and patterns among naturalized rainbow trout (Oncorhynchus mykiss) in two Patagonian lakes differentially impacted by trout aquaculture
Source: Ecol Evol. 2017 Nov 28;8(1):273–85. doi: 10.1002/ece3.3574 (PMC5756871; doi:10.1002/ece3.3574)
Supplement: Supplementary file 1 [file ECE3-8-273-s001.docx]

Table S1. List of SNP loci used in the study. Linkage information between loci is emphasized using lower case letters.

| Locus No | Locus Name | Final SNP panel | Linkage information | Reference |
| --- | --- | --- | --- | --- |
| 1 | *OMS00003* | Included |  | 1,2 |
| 2 | *OMS00006* | Included |  | 1,2 |
| 3 | *OMS00007* | Included |  | 1,2 |
| 4 | *OMS00014* | Included |  | 1,2 |
| 5 | *OMS00018* | Included |  | 1,2 |
| 6 | *OMS00029* | Included |  | 1,2 |
| 7 | *OMS00040* | Included |  | 1,2 |
| 8 | *OMS00041* | Included |  | 1,2 |
| 9 | *OMS00055* | Included |  | 1,2 |
| 10 | *OMS00056* | Included |  | 1,2 |
| 11 | *OMS00057* | Included |  | 1,2 |
| 12 | *OMS00061* | Included |  | 1,2 |
| 13 | *OMS00066* | Included |  | 1,2 |
| 14 | *OMS00067* | Included |  | 1,2 |
| 15 | *OMS00071* | Included |  | 1,2 |
| 16 | *OMS00072* | Included |  | 1,2 |
| 17 | *OMS00074* | Included |  | 1,2 |
| 18 | *OMS00078* | Included |  | 1,2 |
| 19 | *OMS00081* | Included |  | 1,2 |
| 20 | *OMS00087* | Included |  | 1,2 |
| 21 | *OMS00089* | Included |  | 1,2 |
| 22 | *OMS00092* | Included |  | 1,2 |
| 23 | *OMS00105* | Included |  | 1,2 |
| 24 | *OMS00106* | Included |  | 1,2 |
| 25 | *OMS00110* | Included |  | 1,2 |
| 26 | *OMS00116* | Included |  | 1,2 |
| 27 | *OMS00119* | Included |  | 1,2 |
| 28 | *OMS00120* | Included |  | 1,2 |
| 29 | *OMS00121* | Included |  | 1,2 |
| 30 | *OMS00134* | Included |  | 1,2 |
| 31 | *OMS00140* | Included |  | 1,2 |
| 32 | *OMS00153* | Included |  | 1,2 |
| 33 | *OMS00154* | Included | d | 1,2 |
| 34 | *OMS00159* | Included |  | 1,2 |
| 35 | *OMS00164* | Included |  | 1,2 |
| 36 | *OMS00175* | Included |  | 1,2 |
| 37 | *OMS00176* | Included |  | 1,2 |
| 38 | *OMS00180* | Included |  | 1,2 |
| 39 | *Omy_09AAD.076* | Included |  | 1,2 |
| 40 | *Omy_1004* | Included |  | 1,2 |
| 41 | *Omy_101554.306* | Included |  | 1,2 |
| 42 | *Omy_102420.634* | Included |  | 1,2 |
| 43 | *Omy_104519.624* | Included |  | 1,2 |
| 44 | *Omy_105075.162* | Included |  | 1,2 |
| 45 | *Omy_105401.363* | Included |  | 1,2 |
| 46 | *Omy_107336.170* | Included |  | 1,2 |
| 47 | *Omy_107806.34* | Included |  | 1,2 |
| 48 | *Omy_108007.193* | Included |  | 1,2 |
| 49 | *Omy_109243.222* | Included |  | 1,2 |
| 50 | *Omy_110064.419* | Included |  | 1,2 |
| 51 | *Omy_110078.294* | Included |  | 1,2 |
| 52 | *Omy_111005.159* | Included |  | 1,2 |
| 53 | *Omy_111383.51* | Included |  | 1,2 |
| 54 | *Omy_111666.301* | Included |  | 1,2 |
| 55 | *Omy_112301.202* | Included |  | 1,2 |
| 56 | *Omy_112820.82* | Included | c | 1,2 |
| 57 | *Omy_116733.349* | Included |  | 1,2 |
| 58 | *Omy_117259.96* | Included |  | 1,2 |
| 59 | *Omy_117815.81* | Included |  | 1,2 |
| 60 | *Omy_118205.116* | Included | e | 1,2 |
| 61 | *Omy_121713.115* | Included |  | 1,2 |
| 62 | *Omy_127236.583* | Included | a | 1,2 |
| 63 | *Omy_97077.73* | Included |  | 1,2 |
| 64 | *Omy_97954.618* | Included |  | 1,2 |
| 65 | *Omy_aspAT.123* | Included |  | 1,2 |
| 66 | *Omy_cd59.206* | Included |  | 1,2 |
| 67 | *Omy_cxcr.169* | Included |  | 1,2 |
| 68 | *Omy_DABc* | Included |  | 1,2 |
| 69 | *Omy_g12.82* | Included |  | 1,2 |
| 70 | *Omy_gluR.79* | Included |  | 1,2 |
| 71 | *Omy_hsc715.80* | Included |  | 1,2 |
| 72 | *Omy_hsp47.86* | Included |  | 1,2 |
| 73 | *Omy_hsp90BA.229* | Included |  | 1,2 |
| 74 | *Omy_Il.1b.028* | Included |  | 1,2 |
| 75 | *Omy_IL17.185* | Included |  | 1,2 |
| 76 | *Omy_IL1b.163* | Included |  | 1,2 |
| 77 | *Omy_mapK3.103* | Included |  | 1,2 |
| 78 | *Omy_metA.161* | Included |  | 1,2 |
| 79 | *Omy_metB.138* | Included |  | 1,2 |
| 80 | *Omy_nkef.241* | Included | b | 1,2 |
| 81 | *Omy_oxct.85* | Included |  | 1,2 |
| 82 | *Omy_star.206* | Included |  | 1,2 |
| 83 | *Omy_stat3.273* | Included |  | 1,2 |
| 84 | *Omy_tgfb.207* | Included | e | 1,2 |
| 85 | *Omy_u07.79.166* | Included |  | 1,2 |
| 86 | *Omy_u09.56.073* | Included |  | 1,2 |
| 87 | *Omy_mcsf_268-A1* | Excluded - HWE deviation |  | 1,2 |
| 88 | *OMS00012* | Excluded - in LD | c | 1,2 |
| 89 | *Omy_arp-630* | Excluded - in LD | d | 1,2 |
| 90 | *Omy_nkef-308* | Excluded - in LD | b | 1,2 |
| 91 | *Omy_dacd1-131* | Excluded - in LD | a | 1,2 |
| 92 | *Omy_U11_2a-114* | Excluded - in LD | a | 1,2 |
| 93 | *OMS00177* | Excluded - in LD | a | 1,2 |
| 94 | *Ocl_Okerca* | Excluded - monomorphic |  | 3 |
| 95 | *Ocl_oku202* | Excluded - monomorphic |  | 3 |
| 96 | *Ocl_Oku216* | Excluded - monomorphic |  | 3 |

Linkage relationships

a. Group of 4 SNPs separated 3000 to 8000 bp from each other following BLAST of SNPs against whole genome sequencing contigs from rainbow trout (4).

b. Two SNPs with known physical linkage (67 bp apart) from (2).

c. Two SNPs with known linkage map (3.2 cM apart) from (5).

d. Two SNPs separated 1000 to 3000 bp (multiple matches) following BLAST of SNPs against whole genome sequencing contigs from rainbow trout (4).

e. Linkage information unknown as there is no sequence information for Omy_tgfb-207 (unpublished).

References

1. Jones, M. H., et al. (2015). "Consequences of emergence timing for the growth and relative survival of Steelhead Trout fry from naturally spawning wild and hatchery parents." TRANSACTIONS OF THE AMERICAN FISHERIES SOCIETY in press

2. Limborg et al. (2012). "Signatures of natural selection among lineages and habitats in Oncorhynchus mykiss." Ecology and Evolution 2(1): 1-18

3. McGlauflin, M. T., et al. (2010). "High-Resolution Melting Analysis for the Discovery of Novel Single-Nucleotide Polymorphisms in Rainbow and Cutthroat Trout for Species Identification." 2( TRANSACTIONS OF THE AMERICAN FISHERIES SOCIETY 139(3): 676-684.

4. Berthelot et al. (2014). "The rainbow trout genome provides novel insights into evolution after whole-genome duplication in vertebrates." Nat Comm 5

5. Ostberg et al. (2013). "Chromosome rearrangements, recombination suppression, and limited segregation distortion in hybrids between Yellowstone cutthroat trout (Oncorhynchus clarkii bouvieri) and rainbow trout (O. mykiss)." Bmc Genomics 14(1): 570
