# Supplementary material for: Contrasting genetic metrics and patterns among naturalized rainbow trout (Oncorhynchus mykiss) in two Patagonian lakes differentially impacted by trout aquaculture
Source: Ecol Evol. 2017 Nov 28;8(1):273–85. doi: 10.1002/ece3.3574 (PMC5756871; doi:10.1002/ece3.3574)
Supplement: Supplementary file 2 [file ECE3-8-273-s002.docx]

Table S2. Comparison of genetic diversity parameters between this study and Jones et al. (2015).

|  | **This study** | | | | |  | **Jones et al. (2015)** | | | | |  |
| --- | --- | --- | --- | --- | --- | --- | --- | --- | --- | --- | --- | --- |
|  | **Llanquihue** | |  | **TLS** | |  | **Wild** | |  | **Hatchery** | |  |
| **Locus** | **Ho** | **He** |  | **Ho** | **He** |  | **Ho** | **He** |  | **Ho** | **He** | **Ref** |
| OMS00003 | 0.132 | 0.129 |  | 0.010 | 0.010 |  | 0.500 | 0.432 |  | 0.489 | 0.460 | a |
| OMS00006 | 0.456 | 0.494 |  | 0.417 | 0.441 |  | 0.446 | 0.474 |  | 0.289 | 0.333 | a |
| OMS00007 | 0.040 | 0.039 |  | 0.000 | 0.000 |  | 0.022 | 0.021 |  | 0.033 | 0.033 | a |
| OMS00014 | 0.250 | 0.256 |  | 0.002 | 0.002 |  | 0.043 | 0.043 |  | 0.103 | 0.098 | a |
| OMS00018 | 0.173 | 0.175 |  | 0.000 | 0.003 |  | 0.315 | 0.370 |  | 0.211 | 0.239 | a |
| OMS00029 | 0.552 | 0.499 |  | 0.342 | 0.343 |  | 0.054 | 0.072 |  | 0.022 | 0.022 | a |
| OMS00040 | 0.494 | 0.496 |  | 0.528 | 0.471 |  | 0.226 | 0.233 |  | 0.056 | 0.054 | a |
| OMS00041 | 0.512 | 0.474 |  | 0.500 | 0.486 |  | - | - |  | - | - | - |
| OMS00055 | 0.149 | 0.150 |  | 0.495 | 0.495 |  | 0.097 | 0.092 |  | 0.022 | 0.022 | a |
| OMS00056 | 0.450 | 0.474 |  | 0.341 | 0.331 |  | 0.312 | 0.319 |  | 0.389 | 0.386 | a |
| OMS00057 | 0.504 | 0.486 |  | 0.271 | 0.282 |  | 0.355 | 0.481 |  | 0.511 | 0.475 | a |
| OMS00061 | 0.214 | 0.225 |  | 0.349 | 0.332 |  | 0.387 | 0.383 |  | 0.375 | 0.391 | a |
| OMS00066 | 0.298 | 0.301 |  | 0.364 | 0.402 |  | 0.194 | 0.175 |  | 0.233 | 0.255 | a |
| OMS00067 | 0.009 | 0.009 |  | 0.000 | 0.000 |  | 0.000 | 0.000 |  | 0.056 | 0.054 | a |
| OMS00071 | 0.375 | 0.386 |  | 0.134 | 0.133 |  | 0.441 | 0.477 |  | 0.404 | 0.414 | a |
| OMS00072 | 0.438 | 0.459 |  | 0.523 | 0.495 |  | 0.538 | 0.493 |  | 0.393 | 0.457 | a |
| OMS00074 | 0.087 | 0.093 |  | 0.000 | 0.000 |  | 0.473 | 0.497 |  | 0.533 | 0.500 | a |
| OMS00078 | 0.467 | 0.497 |  | 0.511 | 0.499 |  | 0.13 | 0.177 |  | 0.267 | 0.278 | a |
| OMS00081 | 0.295 | 0.369 |  | 0.036 | 0.038 |  | 0.000 | 0.000 |  | 0.022 | 0.022 | a |
| OMS00087 | 0.061 | 0.059 |  | 0.044 | 0.043 |  | 0.247 | 0.217 |  | 0.056 | 0.075 | a |
| OMS00089 | 0.298 | 0.289 |  | 0.091 | 0.090 |  | 0.391 | 0.405 |  | 0.416 | 0.399 | a |
| OMS00092 | 0.201 | 0.195 |  | 0.161 | 0.156 |  | 0.28 | 0.361 |  | 0.156 | 0.143 | a |
| OMS00105 | 0.289 | 0.300 |  | 0.380 | 0.427 |  | 0.323 | 0.383 |  | 0.333 | 0.401 | a |
| OMS00106 | 0.483 | 0.477 |  | 0.005 | 0.005 |  | 0.215 | 0.271 |  | 0.289 | 0.292 | a |
| OMS00110 | 0.331 | 0.357 |  | 0.130 | 0.130 |  | 0.011 | 0.011 |  | 0.056 | 0.054 | a |
| OMS00116 | 0.469 | 0.426 |  | 0.319 | 0.314 |  | 0.108 | 0.121 |  | 0.078 | 0.095 | a |
| OMS00119 | 0.390 | 0.433 |  | 0.512 | 0.499 |  | 0.183 | 0.217 |  | 0.244 | 0.292 | a |
| OMS00120 | 0.377 | 0.389 |  | 0.295 | 0.282 |  | 0.355 | 0.355 |  | 0.478 | 0.448 | a |
| OMS00121 | 0.419 | 0.414 |  | 0.218 | 0.214 |  | 0.522 | 0.500 |  | 0.478 | 0.478 | a |
| OMS00134 | 0.514 | 0.500 |  | 0.412 | 0.438 |  | 0.108 | 0.157 |  | 0.122 | 0.115 | a |
| OMS00140 | 0.479 | 0.480 |  | 0.468 | 0.483 |  | 0.032 | 0.032 |  | 0.000 | 0.000 | a |
| OMS00153 | 0.137 | 0.140 |  | 0.000 | 0.000 |  | 0.330 | 0.304 |  | 0.156 | 0.162 | a |
| OMS00154 | 0.466 | 0.470 |  | 0.340 | 0.351 |  | 0.430 | 0.499 |  | 0.500 | 0.498 | a |
| OMS00159 | 0.326 | 0.324 |  | 0.021 | 0.024 |  | 0.022 | 0.021 |  | 0.011 | 0.011 | a |
| OMS00164 | 0.483 | 0.495 |  | 0.150 | 0.147 |  | 0.269 | 0.248 |  | 0.156 | 0.198 | a |
| OMS00175 | 0.281 | 0.292 |  | 0.282 | 0.306 |  | 0.29 | 0.344 |  | 0.422 | 0.47 | a |
| OMS00176 | 0.455 | 0.454 |  | 0.423 | 0.421 |  | 0.237 | 0.225 |  | 0.256 | 0.27 | a |
| OMS00180 | 0.058 | 0.060 |  | 0.000 | 0.000 |  | 0.326 | 0.34 |  | 0.189 | 0.189 | a |
| Omy_09AAD.076 | 0.482 | 0.489 |  | 0.236 | 0.235 |  | 0.151 | 0.175 |  | 0.056 | 0.054 | b |
| Omy_1004 | 0.443 | 0.446 |  | 0.448 | 0.471 |  | 0.462 | 0.417 |  | 0.344 | 0.478 | c |
| Omy_101554.306 | 0.453 | 0.438 |  | 0.357 | 0.327 |  | 0.129 | 0.121 |  | 0.133 | 0.124 | d |
| Omy_102420.634 | 0.435 | 0.437 |  | 0.345 | 0.332 |  | 0.407 | 0.465 |  | 0.352 | 0.369 | d |
| Omy_104519.624 | 0.443 | 0.431 |  | 0.425 | 0.483 |  | 0.376 | 0.388 |  | 0.467 | 0.464 | d |
| Omy_105075.162 | 0.469 | 0.461 |  | 0.208 | 0.214 |  | 0.489 | 0.444 |  | 0.483 | 0.435 | d |
| Omy_105401.363 | 0.398 | 0.421 |  | 0.490 | 0.500 |  | 0.022 | 0.021 |  | 0.000 | 0.000 | d |
| Omy_107336.170 | 0.395 | 0.382 |  | 0.427 | 0.427 |  | 0.065 | 0.062 |  | 0.078 | 0.075 | d |
| Omy_107806.34 | 0.494 | 0.499 |  | 0.473 | 0.482 |  | 0.398 | 0.496 |  | 0.522 | 0.448 | d |
| Omy_108007.193 | 0.167 | 0.156 |  | 0.000 | 0.000 |  | 0.097 | 0.092 |  | 0.256 | 0.255 | d |
| Omy_109243.222 | 0.259 | 0.261 |  | 0.238 | 0.255 |  | 0.489 | 0.450 |  | 0.422 | 0.499 | d |
| Omy_110064.419 | 0.301 | 0.377 |  | 0.270 | 0.314 |  | 0.204 | 0.200 |  | 0.236 | 0.241 | d |
| Omy_110078.294 | 0.393 | 0.380 |  | 0.018 | 0.018 |  | 0.516 | 0.497 |  | 0.46 | 0.428 | d |
| Omy_111005.159 | 0.153 | 0.153 |  | 0.002 | 0.002 |  | 0.366 | 0.350 |  | 0.261 | 0.260 | d |
| Omy_111383.51 | 0.462 | 0.458 |  | 0.267 | 0.270 |  | 0.484 | 0.441 |  | 0.364 | 0.351 | d |
| Omy_111666.301 | 0.479 | 0.495 |  | 0.523 | 0.495 |  | 0.097 | 0.130 |  | 0.111 | 0.105 | d |
| Omy_112301.202 | 0.152 | 0.161 |  | 0.090 | 0.089 |  | 0.419 | 0.467 |  | 0.511 | 0.500 | d |
| Omy_112820.82 | 0.491 | 0.496 |  | 0.216 | 0.208 |  | 0.217 | 0.243 |  | 0.200 | 0.278 | d |
| Omy_116733.349 | 0.461 | 0.455 |  | 0.440 | 0.441 |  | 0.161 | 0.166 |  | 0.156 | 0.143 | d |
| Omy_117259.96 | 0.234 | 0.235 |  | 0.281 | 0.305 |  | 0.355 | 0.388 |  | 0.422 | 0.437 | d |
| Omy_117815.81 | 0.423 | 0.421 |  | 0.033 | 0.032 |  | 0.409 | 0.412 |  | 0.433 | 0.473 | d |
| Omy_118205.116 | 0.473 | 0.496 |  | 0.490 | 0.472 |  | 0.326 | 0.315 |  | 0.244 | 0.231 | d |
| Omy_121713.115 | 0.216 | 0.209 |  | 0.513 | 0.500 |  | 0.366 | 0.403 |  | 0.289 | 0.320 | d |
| Omy_127236.583 | 0.233 | 0.227 |  | 0.241 | 0.330 |  | 0.269 | 0.292 |  | 0.067 | 0.064 | d |
| Omy_97077.73 | 0.028 | 0.027 |  | 0.065 | 0.069 |  | 0.269 | 0.248 |  | 0.356 | 0.369 | d |
| Omy_97954.618 | 0.450 | 0.492 |  | 0.313 | 0.328 |  | 0.228 | 0.28 |  | 0.384 | 0.386 | d |
| Omy_aspAT.123 | 0.313 | 0.326 |  | 0.459 | 0.472 |  | 0.097 | 0.111 |  | 0.056 | 0.054 | e |
| Omy_cd59.206 | 0.256 | 0.251 |  | 0.479 | 0.463 |  | 0.484 | 0.455 |  | 0.456 | 0.448 | f |
| Omy_cxcr.169 | 0.163 | 0.173 |  | 0.043 | 0.045 |  | 0.011 | 0.011 |  | 0.000 | 0.000 | f |
| Omy_DABc | 0.103 | 0.101 |  | 0.301 | 0.293 |  | 0.065 | 0.062 |  | 0.056 | 0.054 | c |
| Omy_g12.82 | 0.463 | 0.454 |  | 0.405 | 0.383 |  | 0.484 | 0.477 |  | 0.523 | 0.498 | f |
| Omy_gluR.79 | 0.203 | 0.210 |  | 0.494 | 0.500 |  | 0.366 | 0.393 |  | 0.556 | 0.494 | g |
| Omy_hsc715.80 | 0.054 | 0.056 |  | 0.000 | 0.000 |  | 0.402 | 0.443 |  | 0.483 | 0.491 | i |
| Omy_hsp47.86 | 0.152 | 0.161 |  | 0.016 | 0.016 |  | 0.387 | 0.421 |  | 0.344 | 0.352 | i |
| Omy_hsp90BA.229 | 0.162 | 0.163 |  | 0.049 | 0.048 |  | 0.435 | 0.415 |  | 0.3 | 0.27 | i |
| Omy_Il.1b.028 | 0.184 | 0.190 |  | 0.000 | 0.000 |  | 0.538 | 0.497 |  | 0.551 | 0.494 | b |
| Omy_IL17.185 | 0.424 | 0.464 |  | 0.287 | 0.286 |  | 0.538 | 0.458 |  | 0.289 | 0.346 | f |
| Omy_IL1b.163 | 0.111 | 0.114 |  | 0.000 | 0.000 |  | 0.304 | 0.34 |  | 0.416 | 0.378 | f |
| Omy_mapK3.103 | 0.485 | 0.470 |  | 0.428 | 0.428 |  | 0.25 | 0.266 |  | 0.455 | 0.386 | g |
| Omy_metA.161 | 0.395 | 0.414 |  | 0.166 | 0.153 |  | 0.344 | 0.499 |  | 0.438 | 0.464 | g |
| Omy_metB.138 | 0.286 | 0.307 |  | 0.205 | 0.192 |  | 0.204 | 0.2 |  | 0.122 | 0.115 | g |
| Omy_nkef.241 | 0.049 | 0.047 |  | 0.000 | 0.000 |  | 0.435 | 0.423 |  | 0.533 | 0.5 | e |
| Omy_oxct.85 | 0.253 | 0.269 |  | 0.156 | 0.155 |  | 0.312 | 0.331 |  | 0.333 | 0.401 | f |
| Omy_star.206 | 0.518 | 0.482 |  | 0.459 | 0.438 |  | 0.527 | 0.461 |  | 0.422 | 0.444 | f |
| Omy_stat3.273 | 0.487 | 0.498 |  | 0.473 | 0.499 |  | 0.462 | 0.408 |  | 0.467 | 0.494 | f |
| Omy_tgfb.207 | 0.496 | 0.483 |  | 0.500 | 0.498 |  | 0.452 | 0.421 |  | 0.467 | 0.437 | f |
| Omy_u07.79.166 | 0.511 | 0.493 |  | 0.489 | 0.498 |  | 0.527 | 0.498 |  | 0.427 | 0.497 | b |
| Omy_u09.56.073 | 0.235 | 0.226 |  | 0.054 | 0.052 |  | 0.215 | 0.271 |  | 0.326 | 0.316 | b |
|  |  |  |  |  |  |  |  |  |  |  |  |  |
| Mean | 0.327 | 0.331 |  | 0.255 | 0.257 |  | 0.292 | 0.301 |  | 0.287 | 0.292 |  |

References for the markers: a = Sánchez et al. 2009; b = Limborg et al. 2012; c = Hansen et al. 2011; d = AbadíaCardoso et al. 2011; e = Campbell and Narum 2009; f = J. DeKoning, Washington State University, unpublished data; g = N. Campbell, Columbia River Intertribal Fish Commission, unpublished data; h = Stephens et al. 2009; and i = Campbell et al. 2009.
