# Supplementary material for: Contrasting genetic metrics and patterns among naturalized rainbow trout (Oncorhynchus mykiss) in two Patagonian lakes differentially impacted by trout aquaculture
Source: Ecol Evol. 2017 Nov 28;8(1):273–85. doi: 10.1002/ece3.3574 (PMC5756871; doi:10.1002/ece3.3574)
Supplement: Supplementary file 3 [file ECE3-8-273-s003.docx]

Table S3. Pairwise genetic distances (θ) among collections based on the entire dataset. Largest differentiation values were found between lakes (θ > 0.1, in bold).

|  | 1 | 2 | 3 | 4 | 5 | 6 | 7 | 8 | 9 | 10 | 11 | 12 | 13 | 14 | 15 | 16 | 17 | 18 | 19 | 20 | 21 | 22 |
| --- | --- | --- | --- | --- | --- | --- | --- | --- | --- | --- | --- | --- | --- | --- | --- | --- | --- | --- | --- | --- | --- | --- |
| 1. PES13W |  |  |  |  |  |  |  |  |  |  |  |  |  |  |  |  |  |  |  |  |  |  |
| 2. PES14S | 0.002 |  |  |  |  |  |  |  |  |  |  |  |  |  |  |  |  |  |  |  |  |  |
| 3. BLA13W | 0.004 | 0.003 |  |  |  |  |  |  |  |  |  |  |  |  |  |  |  |  |  |  |  |  |
| 4. BLA14S | 0.008 | 0.003 | 0.001 |  |  |  |  |  |  |  |  |  |  |  |  |  |  |  |  |  |  |  |
| 5. TEP13W | 0.011 | 0.005 | 0.007 | 0.005 |  |  |  |  |  |  |  |  |  |  |  |  |  |  |  |  |  |  |
| 6. TEP14S | 0.004 | 0.002 | 0.004 | 0.005 | 0.005 |  |  |  |  |  |  |  |  |  |  |  |  |  |  |  |  |  |
| 7. BAR13W | 0.006 | 0.004 | 0.007 | 0.005 | 0.007 | 0.005 |  |  |  |  |  |  |  |  |  |  |  |  |  |  |  |  |
| 8. BAR13S | 0.011 | -0.001 | 0.009 | -0.003 | 0.007 | 0.005 | -0.022 |  |  |  |  |  |  |  |  |  |  |  |  |  |  |  |
| 9. BAR14S | 0.009 | 0.004 | 0.004 | 0.005 | 0.008 | 0.008 | 0.008 | 0.016 |  |  |  |  |  |  |  |  |  |  |  |  |  |  |
| 10. LEO13W | **0.134** | **0.142** | **0.133** | **0.139** | **0.134** | **0.141** | **0.138** | **0.152** | **0.14** |  |  |  |  |  |  |  |  |  |  |  |  |  |
| 11. LEO14W | **0.112** | **0.115** | **0.108** | **0.114** | **0.109** | **0.116** | **0.111** | **0.128** | **0.115** | 0.015 |  |  |  |  |  |  |  |  |  |  |  |  |
| 12. LEO14S | **0.122** | **0.127** | **0.115** | **0.122** | **0.118** | **0.126** | **0.12** | **0.132** | **0.125** | 0.012 | 0.002 |  |  |  |  |  |  |  |  |  |  |  |
| 13. ESC13W | **0.115** | **0.117** | **0.107** | **0.109** | **0.107** | **0.12** | **0.116** | **0.123** | **0.119** | 0.018 | 0.023 | 0.02 |  |  |  |  |  |  |  |  |  |  |
| 14. ESC14W | **0.109** | **0.112** | **0.103** | **0.106** | **0.106** | **0.12** | **0.108** | **0.124** | **0.115** | 0.023 | 0.027 | 0.029 | -0.001 |  |  |  |  |  |  |  |  |  |
| 15. ESC13S | **0.111** | **0.109** | **0.105** | **0.103** | **0.102** | **0.116** | **0.105** | **0.104** | **0.111** | 0.015 | 0.022 | 0.019 | 0.007 | 0.009 |  |  |  |  |  |  |  |  |
| 16. ESC14S | **0.131** | **0.135** | **0.125** | **0.125** | **0.123** | **0.138** | **0.127** | **0.128** | **0.133** | 0.019 | 0.031 | 0.021 | 0.012 | 0.012 | -0.001 |  |  |  |  |  |  |  |
| 17. CCH13W | **0.127** | **0.13** | **0.121** | **0.125** | **0.121** | **0.133** | **0.131** | **0.136** | **0.128** | 0.017 | 0.03 | 0.022 | 0.005 | 0.006 | 0.012 | 0.017 |  |  |  |  |  |  |
| 18. CCH14S | **0.119** | **0.125** | **0.116** | **0.12** | **0.115** | **0.128** | **0.125** | **0.131** | **0.122** | 0.017 | 0.025 | 0.023 | 0.003 | 0.009 | 0.009 | 0.014 | 0.002 |  |  |  |  |  |
| 19. CAY13W | **0.149** | **0.146** | **0.143** | **0.143** | **0.141** | **0.152** | **0.145** | **0.156** | **0.148** | 0.029 | 0.063 | 0.046 | 0.028 | 0.042 | 0.031 | 0.029 | 0.019 | 0.026 |  |  |  |  |
| 20. CAY13S | **0.115** | **0.113** | **0.111** | **0.112** | **0.11** | **0.124** | **0.109** | **0.111** | **0.106** | 0.024 | 0.02 | 0.015 | 0.025 | 0.038 | 0.022 | 0.026 | 0.007 | 0.011 | -0.02 |  |  |  |
| 21. PEU14W | **0.126** | **0.126** | **0.122** | **0.125** | **0.12** | **0.134** | **0.122** | **0.142** | **0.125** | 0.02 | 0.043 | 0.038 | 0.01 | 0.023 | 0.022 | 0.025 | 0.005 | 0.008 | 0.011 | 0.019 |  |  |
| 22. PEU14S | **0.12** | **0.12** | **0.115** | **0.117** | **0.114** | **0.125** | **0.121** | **0.143** | **0.12** | 0.024 | 0.035 | 0.031 | 0.009 | 0.019 | 0.028 | 0.03 | 0.009 | 0.01 | 0.016 | 0.011 | 0 |  |
